# Supplementary material for: Prenatal development of neonatal vocalizations
Source: eLife. 2022 Jul 26;11:e78485. doi: 10.7554/eLife.78485 (PMC9391037; doi:10.7554/eLife.78485)
Supplement: Supplementary file 1. [file elife-78485-supp1.docx]

***Supplementary materials***

**The statistical results for individuals (related to Figures 4C and 4I)**

The sample size for each pregnancy is small (limited naturally by the gestation period of the marmoset). Though we did see significance at the population level--even after controlling for pregnancies--we did not have enough statistical power for individual pregnancies. Nevertheless, we report those results here. Importantly, the sign of the effect (β) is consistent for each pregnancy.

Matching temporal profiles of fetal orofacial movements to that of week 1 infant contact calls (related to Figure 4C)

Pregnancy 1 β±SE = -0.01±0.01, t = -1.28; F(1,10)=1.64; p=.23

Pregnancy 2 β±SE = -0.003±0.002, t = -1.38; F(1,15)=1.90; p=.19

Pregnancy 3 β±SE = -0.008±0.007, t = -1.11; F(1,14)=1.23; p=.29

Pregnancy 4 β±SE = -0.003±0.005, t = -0.53; F(1,14)=0.28; p=.60

Matching fetal orofacial movements to infant call profile (related to Figure 4I)

Pregnancy 1 β±SE = 0.03±0.13, t = 0.25; F(1,13)=0.06; p=.80

Pregnancy 2 β±SE = 0.27±0.08, t = 3.22; F(1,16)=10.38; p=.006

Pregnancy 3 β±SE = 0.18±0.08, t = 2.21; F(1,16)=4.89; p=.04

Pregnancy 4 β±SE = 0.08±0.06, t = 1.17; F(1,15)=1.36; p=.26
